# The Effect of Macronutrients on Reproductive Hormones in Overweight and Obese Men: A Pilot Study

**DOI:** 10.3390/nu11123059

**Published:** 2019-12-14

**Authors:** Karma L. Pearce, Kelton Tremellen

**Affiliations:** 1 School of Pharmacy and Medical Sciences, ARENA, University of South Australia, Adelaide 5001, South Australia, Australia; Ktremellen@repromed.com.au; 2 Repromed, Dulwich 5065, South Australia, Australia; 3 Department of Obstetrics Gynaecology and Reproductive Medicine, Flinders University, Bedford Park 5042, South Australia, Australia

**Keywords:** macronutrients, protein, carbohydrates, lipids, testosterone, overweight, obese, men, oestradiol, follicle stimulating hormone (FSH), luteinising hormone (LH)

## Abstract

Hypogonadal obese men find it difficult to lose weight. We investigated whether the modification of macronutrient intake can alter testosterone levels independently of the body mass index. Fasted overweight or obese fertile men were asked to consume meals of polyunsaturated fats (PUFA), monounsaturated fats (MUFA), refined carbohydrates (CHO, orange juice, OJ), whey and egg albumin and mixed meals of PUFA and CHO, PUFA and egg albumin, and CHO and egg albumin. Blood was collected at fasting, then hourly for 5 h and analysed to determine the levels of testosterone and other hormones. We found PUFA and MUFA or a mixed meal of PUFA and CHO significantly reduced serum testosterone production to a similar degree over a 5 h period. PUFA decreased serum testosterone levels by 3.2 nmol/L after 1 h compared to baseline (*p* = 0.023), with this suppression remaining significant up to 5 h postprandially (2.1 nmol/L; *p* = 0.012). The net overall testosterone levels were reduced by approximately 10 nmol/L × h by PUFA, MUFA and PUFA combined with CHO. CHO alone had little effect on testosterone levels, whereas egg albumin was able to increase them (7.4 cf 2.0 nmol/L × h). Therefore, for men wishing to optimize their testosterone levels, it may be wise to avoid a high fat intake, drink liquids such as water or OJ or even consider fasting. ANZCTR, Australia; ACTRN12617001034325.

## 1. Introduction

Obesity has become a global health problem that is reaching epidemic proportions, with 1.9 billion adults classified as overweight, and an additional 650 million adults classified as obese [1]. In Australia, obesity has nearly tripled in the past 30 years in reproductive-age men [2], with 71% of men currently classified as overweight or obese [3]. This coincides with a significant increase in male infertility in Australia and worldwide [2]; in addition, recent research has also linked male obesity to hypogonadism [4].

Testosterone is the fundamental sex hormone in men and plays significant roles in reproductive and sexual functions [5]. Almost all testosterone is produced by the testicular Leydig’s cells, primarily regulated through the hypothalamic–pituitary–luteinizing hormone (LH) drive [2]. A number of epidemiological studies have shown inverse relationships between total testosterone and obesity (reviewed in [6]), and although obesity is known to be one of the key drivers of androgen deficiency and impaired fertility, the obesity–hypogonadism relationship is thought to be bidirectional, with adiposity having most pronounced effects on the hypothalamic–pituitary (HP) axis [6]. Adipose tissue-derived aromatase converts testosterone to oestradiol, which in turn suppresses the release of LH from the hypothalamus [7]. In parallel, visceral fat harbours increased levels of adipokines, such as leptin which leads to the suppression of LH and subsequent testosterone production [8], and proinflammatory cytokines such as IL-6, IL-17, TNFα and IL-1β, which impair testosterone production by interstitial macrophages residing adjacent to Leydig cells [9]. There is also evidence that inflammatory cytokines impair hypothalamic–pituitary (HP) axis function [8]. Furthermore, our own group has also shown obese men have an increased prevalence of leaky gut (low-grade inflammation induced by the passage of intestinal bacteria-derived lipopolysaccharide (LPS)) [10], and leaky gut and obesity were associated with metabolic endotoxemia (ME) [10]. ME has also been associated with reduced testosterone levels in epidemiological surveys and has proven to be a cause of hypogonadism when LPS is experimentally administered to men [11]. Taken together, these studies provide strong evidence for a link between obesity-driven inflammation and testosterone deficiency. Additionally, meals high in fat and carbohydrates, but not in proteins, [12] are known to be associated with both postprandial ME and inflammation [13]. As such, we postulate that since inflammation and ME are known to play an important role in hypogonadism, different macronutrients may have variable effects on testicular function.

Recent studies illustrate that nutrition plays a critical role in the normal function of the reproductive system. Existing epidemiological studies have demonstrated a link between infertility and dietary patterns [14]. The increasing popularity of saturated fat, trans-fatty acids, sugar and sodium and the lower intakes of antioxidant-rich fruits and vegetables, often characterised as a ‘Western’ dietary pattern, have been associated with reduced reproductive potential [14,15,16]. In contrast, ‘health-conscious’ or ‘prudent’ dietary patterns typically characterised by high intakes of fruits, vegetables, fish and whole grains have been significantly associated with improved reproductive function [16,17]. Furthermore, Mediterranean dietary patterns assessed in a cross-sectional study of 225 men of reproductive age attending a fertility clinic were positively associated with higher sperm concentration, total sperm count and sperm motility, yet, surprisingly, no data on testosterone levels were reported [18].

Typically, obese men with impaired reproductive function have a diet high in saturated fat and refined carbohydrates and low in fresh fruit and vegetables (reviewed in [19]). As such, obesity-related hypogonadism may be a direct result of these dietary macronutrients and micronutrient intakes, rather than just reflecting excess adiposity. One of the largest cross-sectional studies examining the association between male fertility and dietary fat intake demonstrated a clear link between increased total dietary fat intake, in particular saturated fat, and lower sperm quality, thought to be the result of the accumulation of fatty acids within the testicular environment, leading to impaired spermatogenesis and low testosterone synthesis by Leydig cells [14,19,20]. Interestingly, men with the highest energy intake from monounsaturated fat (MUFA) had a lower percentage of morphologically normal spermatozoa [21]. High amounts of sugar-sweetened beverages are also thought to have a detrimental effect on male fertility, although a cross-sectional study of 189 healthy men found sugar-sweetened beverages had little or no effect on serum testosterone levels [22].

The potential biases associated with assessing average food intakes with food frequency questionnaires are well recognised. In addition, the fact that adiposity is the net result of not only the type and amount of food ingested, but also the level of exercise and genetic factors makes an accurate assessment of the influence of macronutrients on testicular function difficult. To overcome these limitations, a small number of researchers have conducted short-term interventional feeding studies to assess the impact of macronutrient intake on testosterone production. While some acute studies have confirmed that meals high in saturated fat produce on average a 30% postprandial reduction in testosterone levels within 1 h of their ingestion, returning to baseline in the next 4–6 h [23,24,25], a single study showed a chronic fat intake decreased testosterone [23]. Another single study showed a mixed meal of carbohydrate and protein did not alter either free or total testosterone concentrations [25]. Yet, to the best of our knowledge, no studies have evaluated the impact of different types of fat (MUFA, polyunsaturated fat (PUFA)) in combination with protein on testosterone production.

As obese men, in particular, find it very difficult to lose weight, especially if they are hypogonadal, which reduces their basal metabolic rate and motivation to exercise, it is vital that we further investigate if modifications of the dietary intake can alter total testosterone levels independent of changes in body mass index (BMI). Our study aimed to investigate in an inpatient setting the impact of various macronutrients of precise content on testicular endocrine function, while controlling for habitual diet and other factors like sleep and exercise levels.

## 2. Materials and Methods

### 2.1. Cohort

Overweight or obese men aged 18 to 50 years were recruited between March 2018 and August 2019 via advertisements and social media. Exclusion criteria were documented inflammatory or infectious disease, primary hypogonadism (Klinefelters Syndrome, cryptorchidism or testicular injury), the consumption of immunosuppressive medication (e.g., nonsteroidal anti-inflammatory drugs (NSAID), corticosteroids or fish oil) or antibiotics and supplements that may alter intestinal function (e.g., probiotics or prebiotics) or any male hormonal therapy (i.e., aromatase inhibitors, clomiphene citrate, human chorionic gonadotropin (hCG) or testosterone). This study was approved by the University of South Australia Ethics Committee (approval number 00000200913), with informed written consent being obtained from all participants. The trial was also prospectively registered with the Australian and New Zealand Clinical Trials Registry (ANZCTR, Camperdown, Australia; ACTRN12617001034325).

### 2.2. Anthropometric Measures

Height was measured to the nearest 1cm (Seca, 216, Germany). Weight was measured using a bio-electrical impedance scale (Tanita, UM051, Tanita Corporation of America Inc., Arlington Heights, IL, USA). BMI was calculated using the equation body weight (kg)/height (m^2^), and the participants were classified using the WHO ranges in overweight (25.00 kg/m^2^–29.99 kg/m^2^) and obese (>30 kg/m^2^) [26].

### 2.3. Habitual Behaviour Patterns

#### 2.3.1. Dietary Intake Data

Following individual instructions from a registered nutritionist, participants weighed their dietary intake for seven consecutive days and recorded the weight, type, brand and amount of foods and beverages consumed. The participant’s food diary was checked by the nutritionist in the presence of the participant to assess the accuracy and completion of the diary. Food models and measuring utensils were used to assist the participants in clarifying the quantities of food and beverages consumed outside their home. The dietary data were entered into Foodworks 8 software (Xyris Software Australia Pty Ltd., Highgate Hill, Australia). Foods not included in the database were entered manually on the basis on provided recipes and food labels. In addition, 20% of the dietary data were cross-checked by another researcher to ensure precision and accuracy. Dietary intake data from 5 weekdays and 2 weekend days were included in the analysis and averaged. To detect reported energy levels that were potential underestimates of actual intake, the basal metabolic rate (BMR) was determined using the Scofield equation [27]. Individualised under-reporting cuff-offs outside of the 95% CI for plausible energy requirements based on the energy intake-to-BMR ratio were determined using the method described by Black [27].

#### 2.3.2. Habitual Sleep and Exercise Patterns

Participants completed the Pittsburgh Sleep Quality Index questionnaire [28] to determine their sleep quality, while average physical activity levels were quantified using the Baecke Physical Activity Questionnaire [29].

### 2.4. Intervention Protocol

#### 2.4.1. Baseline (Fasting) Protocol

After maintaining an overnight fast from food and all beverages except for water, baseline blood samples were collected from all participants between 7:30 and 8:30 on the day of testing. The participants remained fasted over the 5 hours of blood collection, except for water.

#### 2.4.2. Feeding Protocol

After an overnight fast and baseline blood collection, participants were asked to consume food (which varied according to the feeding protocol undertaken) within 15 min of the initial blood draw, over a maximum period of 10 min. The participants continued to have their blood collected hourly over a total of 5 h from baseline. Access to water was unrestricted, with no other food provided over the 5 h period. The nutritional composition of each feeding protocol is displayed in Table 1.

There were 9 feeding protocols: the refined carbohydrate arm (CHO) required participants to consume 1 L of orange juice (OJ, Nudie (nothing but oranges), Melbourne Australia); the protein arm required participants to drink either 52 g of egg white protein powder (Bulk nutrients, Tasmania, Australia) dissolved in 600 mL of water or 47 g of powdered whey protein (Bulk nutrients, Tasmania, Australia) mixed with 600 mL of water; the fat protocols consisted of 250 mL of PUFA (Intralipid^TM^ 20% soybean oil emulsion) or 55 g of MUFA (extra-virgin light olive oil (Moro, Papua New Guinea, Australia). A combined PUFA fat and CHO arm consisted of 1 L of OJ consumed with 250 mL of Intralipid^TM^ 20% soy oil emulsion, whereas a combined PUFA and protein protocol consisted of 250 mL of Intralipid^TM^ 20% soy oil emulsion followed by 52 g of powdered egg white protein (Bulk nutrients, Tasmania, Australia) dissolved in 600 mL of water, and the final feeding protocol consisted of 52 g of powdered egg white protein. The amount of total fat, carbohydrates, and proteins in each of the feeding protocols was designed to match the macronutrients to a McDonalds^TM^ meal of 2 English muffins (sausage, egg, and cheese) and 2 hash browns—a meal that we have previously reported to produce a significant post-prandial fall in testosterone [30].

### 2.5. Statistical Analysis

All demographic and biochemical variables were expressed as a range and mean ± standard deviation. All variables were tested for normality and log-transformed where appropriate. Correlation analysis was performed with SPSS using the Pearson method for normally distributed data and the Spearman method for non-parametric data (IBM Corporation, New York, NY, USA). A two-way ANOVA was used to determine differences between the baseline and the treatment arm of the study at the various time points across the monitoring period. Adjustments were made where appropriate for BMI, age, physical activity levels, sleep, total energy and fat for the habitual diet. Statistical significance was set at 0.05.

## 3. Results

### 3.1. Subjects

A total of nine healthy men aged 34.8 ± 10.6 years, BMI of 31.2 ± 5.1 kg/m^2^ and energy intake 10.3 ± 2.9 Mj/d participated in the study between March 2018 and August 2019. Five participants were recruited for each of the nine feeding protocols. Unfortunately, the individual participants in each feeding protocol varied over the 18-month duration of the study due to participants gaining full time employment (and no longer being available), which made changes in participants necessary.

The demographic, lifestyle and dietary intake data for each feeding protocol are provided in Table 2. There were no significant differences in the demographic, lifestyle factors, energy or macronutrient intakes and baseline hormone status across the fasting or feeding protocols (Table 2 and Table 3).

### 3.2. Hormone Analysis

The biochemical measures in the fasting state for each of the feeding protocols are outlined in Table 3.

#### 3.2.1. Testosterone

Overall, the mean serum testosterone level at baseline was 11.7 ± 3.0 nmol/L. As expected, testosterone was also strongly and negatively correlated with BMI (*p <* 0.001; *r* = −0.571), which was maintained after adjustment.

As there was a significant difference in the baseline (fasting) measures across the feeding protocols for testosterone, oestradiol and follicle stimulating hormone (FSH), the data were normalised and presented as a measure of change from the fasting baseline. Sleep was positively and moderately correlated with fasting testosterone levels (*r* = −0.359, *p =* 0.029). No other relationships were observed between serum testosterone and physical activity or habitual diet.

Dietary intake data were assessed from weighted food records from 5 weekdays and two weekend days using Foodworks 8 software (Xyris Software Australia Pty Ltd., Australia). Data are expressed as mean ± SD.

**Figure 1:**
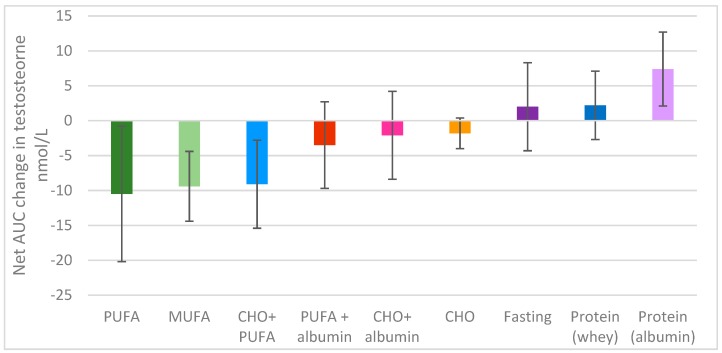
Net area-under-the-curve change in testosterone over 5 h.

There was a significant difference overall in the change in testosterone levels over time between the different feeding protocols (*p <* 0.001); the 5 h time period explained 16% of the variance (*p =* 0 < 0.001), and the different feeding protocols explained a further 17% of the variance, with a feeding protocol x time interaction approaching significance; *p =* 0.053 (Figure 1).

**Figure 2:**
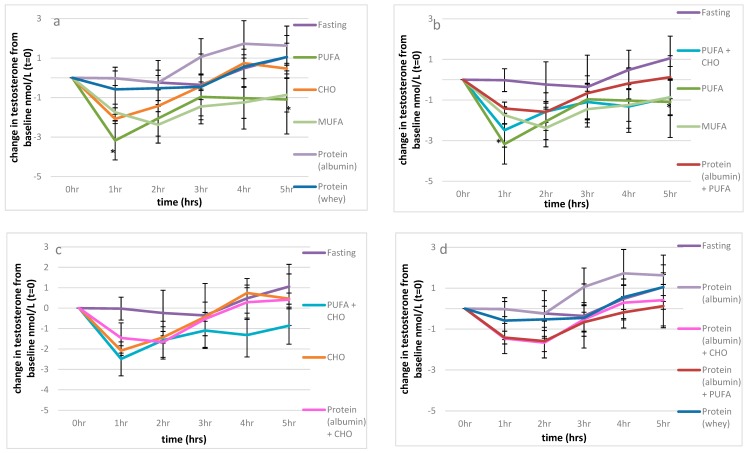
The effect of macronutrients on serum testosterone levels. (**a**): Macronutrients; change from baseline testosterone measures, (**b**): impact of carbohydrates and proteins on fat; change from baseline testosterone measures, (**c**): impact of fat and proteins on refined carbohydrates; change from baseline testosterone measures, (**d**): impact of fat and carbohydrates on proteins; change from baseline testosterone measures. Significance expressed as change from fasting levels; * *p <* 0.05.

Fasting produced minimal changes in serum testosterone levels over the observed 5 h period. However, compared to fasting, there was a significant fall in testosterone levels following the consumption of 52 g of PUFA (*p <* 0.001) and 51 g MUFA (*p =* 0.001) (Figure 1, Figure 2a). In fact, 52 g of PUFA decreased serum testosterone levels by 3.2 nmol/L after 1 h compared to baseline (*p =* 0.023), with this suppression remaining significant up to 5 h post-consumption (1.2 nmol/L; *p =* 0.012) Figure 2a. A similar serve of MUFA (51 g) produced a comparable effect on serum testosterone levels relative to fasting over time (*p =* 0.027) as shown in Figure 2a, reducing the net overall testosterone levels by approximately 10 nmol/L × h for both PUFA and MUFA (Figure 1), which suggests that the type of fat is of great importance for testosterone levels (Figure 1).

Furthermore, refined carbohydrates (1 L of orange juice) were unable to ameliorate the detrimental effect of 52 g PUFA on serum testosterone compared to fasting (*p =* 0.040), as shown in Figure 1 and Figure 2b. Conversely, the addition of 41 g of egg albumin to 52 g PUFA reduced the detrimental effect on testosterone levels, such that there we no longer significant differences over the 5 h period compared to fasting (*p =* 0.611), as shown in Figure 1 and Figure 2b.

Refined carbohydrates (CHO; 1 L orange juice) had no significant impact on serum testosterone levels overall (Figure 1). Additionally, the effects of the combination of 41 g of egg albumin with CHO were similar to that of CHO alone compared to fasting (*p =* 0.927, *p =* 0.834 respectively) (Figure 2c). As previously discussed, the addition of 52 g PUFA to refined carbohydrates (1 L of orange juice) considerably decreased serum testosterone compared to fasting (*p =* 0.040) (Figure 1 and Figure 2b,c).

Surprisingly, the type of protein ingested appeared to differentially impact serum testosterone levels. Pure albumin significantly increased serum testosterone levels over the 5 h period, with the minor postprandial increase observed after whey ingestion not significantly different from value of the fasted state (Figure 1). Importantly, the consumption of 41 g of egg white protein increased serum testosterone levels by almost four-fold relative to fasting (7.4 cf 2.0 nmol/L × hour), with a difference of approximately 17–18 nmol/L x hour relative to either 52 g PUFA, 55 g MUFA or the combination of CHO (1 L orange juice and 52 g PUFA over the 5 h sampling period (Figure 1). Additionally, the combination of CHO (1 L orange juice) with 52 g PUFA (Figure 2c) and the ingestion of 52 g of PUFA alone (Figure 2a,b) had detrimental effects on serum testosterone relative to 41 g of egg white protein 1 h after consumption (*p =* 0.006, *p =* 0.03 respectively), and these effects were maintained for 4 h (*p =* 0.008, *p =* 0.002, respectively) and 5 h post-consumption ((*p =* 0.029, *p =* 0.017, respectively), along with the effect of 55 g of MUFA (*p =* 0.029) (Figure 2d).

#### 3.2.2. LH

**Figure 3:**
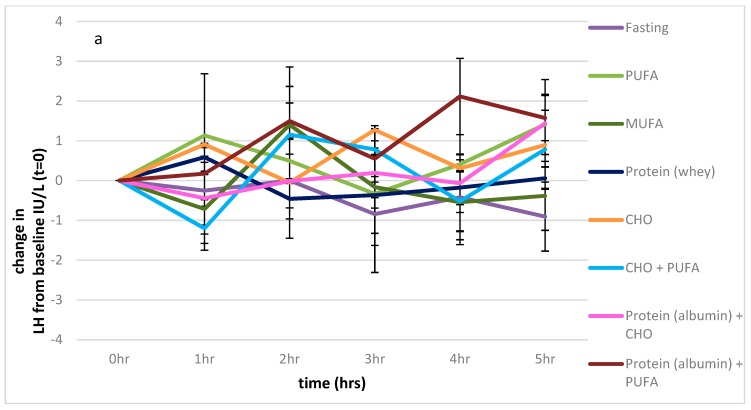

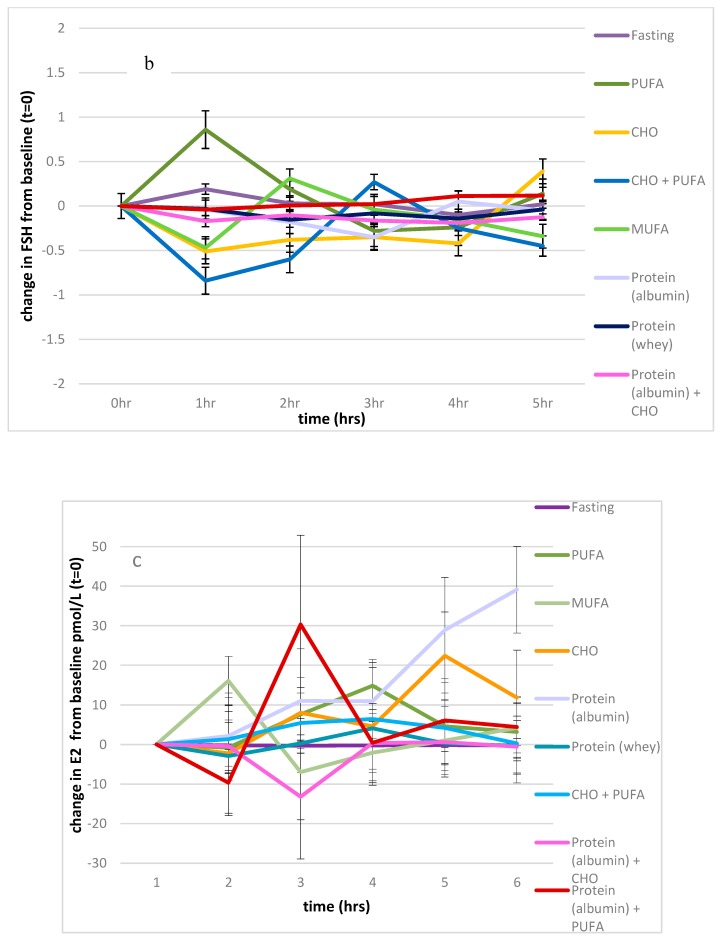
Impact of macronutrients on oestradiol, FSH, and LH. (**a**): Impact of nutrients on change from baseline, LH measures, (**b**): impact of nutrients on change from baseline, FSH measures, (**c**): impact of nutrients on change from baseline, oestradiol. Significance expressed as change from fasting levels; * *p <* 0.05.

There were no significant differences in LH levels between any of the feeding protocols over the 5 h sampling period (*p =* 0.490). (Figure 3a).

#### 3.2.3. FSH

There was also a significant effect of the feeding protocols on FSH levels (*p <* 0.001), which contributed to 25% of the variance, with the effect of time approaching significance (*p =* 0.061; 6% of the variance), and the interaction between the feeding protocol and time also approaching significance (*p =* 0.059). The values of the fasting state were significantly different from those of the PUFA and combined PUFA and CHO feeding protocols (*p =* 0.023, *p =* 0.041 respectively), as shown in Figure 3b.

#### 3.2.4. Oestradiol

There was a significant effect of the feeding protocols on oestradiol (*p <* 0.001), which contributed 27% of the variance over time (*p =* 0.035) and showed an interaction between the feeding protocols and time (*p =* 0.045, 7% of the variance). Compared to the fasting state, there was a significant effect of all feeding protocols on oestradiol levels (*p* > 0.001 for all), except for the feeding protocol consisting of 41 g of egg white powder (*p =* 0.563), as shown in Figure 3c. Serum oestradiol levels significantly increased above the fasting baseline with the albumin protocol.

After consuming 41 g of egg white protein, there were no significant relationships between serum testosterone and oestradiol 4 and 5 h post-consumption, whereas one would have expected an increase in testosterone to be accompanied by an increase in oestradiol due to aromatisation (*p =* 0.588, *p =* 0.960 respectively).

### 3.3. Habitual Dietary Intake Data

The mean habitual dietary pattern was 1.1 ± 0.29 Mj/day, 89 ± 31 g protein, 95 ± 28 g fat, 30 ± 9 g saturated fat, 304 ± 114 g carbohydrates and 26 ± 13 g fibre. There were no significant relationships between any of the macronutrients or fibre and testosterone, LH or FSH levels. Oestradiol levels were positively correlated with energy intake and sugar levels (*r =* 0.538, *p =* 0.001, *r =* 0.678, *p <* 0.001 respectively).

Furthermore, the ratio between habitual dietary protein and carbohydrate intake was not related to serum testosterone levels (*p =* 0.958), but moderately and positively correlated with LH and FSH levels (*p =* 0.016; *r =* 0.327, *p =* 0.016; *r =* 0.326, respectively). These relationships were maintained after adjustment for age and BMI.

## 4. Discussion

Many previous studies have shown a consistent 20%–30% reduction in testosterone levels following a mixed meal. However, for the first time, this interventional study has clearly shown that the nature of macronutrient intake significantly impacts on testosterone production. The first key finding of this study is that fat, in both PUFA and MUFA forms, or a mixed meal of PUFA and CHO significantly reduced serum testosterone production to a similar degree over a 5 h period. We have previously shown that a standard high-fat McDonald’s meal of two English muffins (sausage, egg, and cheese) and two hash browns, containing approximately 50 g of total fat, also decreased serum testosterone production over a 5 h period by 9.8 ± 2.4 nmol/L × h, which was comparable to what observed in this study [29]. We have previously shown that intravenous administration of PUFA (approximately half the amount consumed in this study) had no impact on serum testosterone, LH, oestradiol or FSH, while an identical oral dose of 25 g PUFA suppressed testosterone by approximately half the level observed in this study, implying a dose–response effect. This result suggests that fat does not directly impair Leydig cell function, but rather the passage of fat through the intestinal tract elicits a dose–response inhibition of testosterone production. Given the well-established link between inflammation and impaired testicular function, plus the anti-inflammatory effects of the Mediterranean diet, we postulated that a MUFA meal of olive oil may reduce inflammation and therefore have no negative impact on Leydig cell function. Furthermore, many of the health benefits attributed to MUFA or, more specifically, to extra-virgin olive oil (EVOO), arise from bioactive antioxidants (polyphenols, tocopherols and phytosterols) with postulated anti-inflammatory properties [31]. However, MUFA olive oil had a similar negative impact on serum testosterone levels to that of plant-based PUFA or animal-based fats (McDonald’s study). As the results of this study compared to our previous publications do suggest a dose–response effect, it appears that the dose of fat, not the type of fat, is the primary determinant of the postprandial decline in testosterone production [30].

This study showed that an OJ meal high in CHO had little effect on serum testosterone, with the minor postprandial reduction in testosterone levels not reaching significance compared to that in the fasting state. This finding was a little surprising, as previous studies have linked the ingestion of 75 g of glucose or dextrose to significant falls in testosterone levels [32,33]. It is interesting to speculate why OJ does not significantly impair testosterone production, whereas simple sugar solutions do. One potential mechanism is that OJ contains significant amounts of antioxidants, whereas simple sugar solutions do not. As oxidative stress is known to impair Leydig cell function and antioxidants such as resveratrol have been shown to boost testosterone production [34], it is possible that the CHO content in OJ does suppress testosterone production, but the antioxidants in OJ boost Leydig cell health, limiting that suppression. Of course, future studies comparing the testosterone levels after the administration of simple sugar and OJ loads of identical CHO content will be required, measuring the antioxidant status to prove this theory. Unsurprisingly, the combination of OJ with PUFA failed to ameliorate the postprandial fall in testosterone associated with a PUFA meal (which was similar, overall, to the fall in serum testosterone observed as a result of consumption of a McDonald’s mixed meal [30]). We also have recently observed a fall in serum 8-hydroxydeoxyguanosine (8-OHdG) after the high-fat mixed McDonald’s meal, consistent with antioxidants in the food reducing oxidative damage to DNA, and concluded it was also unlikely that oxidative stress is the underlying cause of the postprandial drop in testosterone [30]. Combined, these findings are supported by a recent study showing that 760 men participating in a National Health and Nutrition Examination Survey (NHANES), who adhered to a Mediterranean-style dietary pattern typically characterised by high intakes of fruits, vegetables, whole grains and fish and a low intake of meat [16,19,35], with 40% of calories from fat (approximately 85 g) and high in antioxidants, possessed significantly lower mean serum testosterone levels compared to men consuming an ad libitum diet (14.3 nmol/L c.f 15.4 nmol/L, respectively). These effects were maintained after controlling confounders of age, BMI, physical activity, comorbidities, diabetes and prostate cancer [36]. Our findings may have implications for the popular Mediterranean dietary pattern which promotes the consumption of 58–67 g of fat each day [31], the majority of which is ‘healthy’ fat. Our results have clearly shown that it is the dose of fat, not its type (MUFA vs PUFA) that impacts on testicular function. As such, the Mediterranean diet is unlikely to have a positive benefit for androgen production. Finally, it is interesting to note that the NHANES database also showed that men adhering to a very low fat diet, similar to that recommended by the American Heart Association (31% of energy from total fat (60–80 g/day)), were also more likely to be hypogonadal than men who consumed an ad libitum diet [36]. As such, it appears that too much and too little dietary fat can negatively impact on testicular function. Future studies should evaluate whether consumption of the Mediterranean diet or ‘healthy-heart’ dietary patterns impairs testosterone production over the long term, as only acute changes were assessed in the current study.

The second key finding of our study is that a pure protein meal of approximately 50 g of protein did not produce a postprandial drop in serum testosterone, as has been reported in other studies using mixed meals with similar protein loads [32]. Most importantly, rather than reducing testosterone, egg albumin significantly improved testosterone levels compared to MUFA, PUFA and a McDonald’s mixed meal (7.4 ± 5.3, −9.4 ± 5.0, −10.5 ± 9.7, −9.8 ± 2.4 nmol/L × h, respectively). Additionally, a mixed meal of egg albumin and PUFA, compared to PUFA consumed alone, was able to blunt the postprandial drop in testosterone by 65% overall, with the largest differences observed within the first, fourth, and fifth hour (22%–48%). We believe that this result most likely represents the net effect of a drop in testosterone production induced by PUFA and a stimulatory effect of albumin.

Although not statistically significant, the type of protein consumed also appears to be important, as whey produced a smaller increase in serum testosterone compared to pure egg albumin. Habito et al. observed differences in male sex hormone concentrations between men consuming soy tofu or meat as part of a habitual diet over a 4-week period; however, the differences were modest [24].

The mechanism by which albumin may boost testosterone production is presently unknown. Protein ingestion is known to have a positive impact on the somatomedin axis, increasing free Insulin-like growth factor-1 IGF-1 levels [37]. As IGF-1 is known to enhance testosterone production by Leydig cells in vitro [38], it is possible that a postprandial boost in serum IGF-1 levels after albumin ingestion is responsible for increasing serum testosterone levels. Future studies examining this possibility are clearly required.

We acknowledge several potential weaknesses in our study. Firstly, we acknowledge the relatively small sample size of our pilot study. This was necessary, due to the high cost of volunteer reimbursement for the invasive in-patient feeding protocol. However, as we found distinctive statistically significant effects on serum testosterone levels by the various feeding protocols, particularly those consisting of total fat and protein, we do not believe this was a substantial limitation. In addition, this study only recruited overweight and obese men. We chose to focus on overweight and obese men for two reasons. It is widely accepted that overweight and obese men are more likely to have metabolic endotoxemia due to increased intestinal permeability and exhibit a more exaggerated inflammatory response to a meal. In addition, many overweight or obese men are likely to be hypogonadal [10,33], and we felt that any postprandial fall in testosterone is more likely to be clinically meaningful in this cohort compared with lean eugonadal men, giving our results more clinical relevance. However, it is highly likely that similar outcomes will be observed in lean men. Indeed, similar postprandial falls in serum testosterone (2.5–4 nmol/L) after feeding have been reported in men with BMIs in the range of 18–2 kg/m^2^ [23,39]. The recruitment of a larger sample size over a wider range of body types is likely to indicate the generalisability of the current findings to the broader population. Finally, we recognise that LH levels fluctuate over the day, and we may have missed small changes by sampling only hourly, rather than more frequently. A refined-CHO meal has been shown to suppress pulsatile LH over a 40 min period, when sampling occurred at 5 min intervals [39]; therefore, we cannot categorically state that the observed drop in testosterone injection induced by fat or refined carbohydrate and fat is related to pituitary suppression or a direct inhibitory effect on Leydig cells. We also acknowledge we could have reported sex hormone-binding globulin (SHBG) and free (bioactive) testosterone levels. However, we believed this to be unnecessary, as a number of other research groups have reported no statistically significant changes in SHBG levels after the consumption of a mixed meal [32], a high-fat meal [20,40] or a refined carbohydrate meal [34].

## 5. Conclusions

In conclusion, it appears that proteins, particularly egg white protein, may be beneficial in enhancing serum testosterone levels, with this effect observed within 4 to 5 h after meal consumption. Conversely, both MUFA “good fat” and PUFA “bad fat” had a similar negative impact on post-prandial testosterone levels over a 5 h period. Carbohydrate intake, at least in the form of highly antioxidant OJ, had very minimal impact on serum testosterone. As such, we would advocate that hypogonadal men, or those with low normal testosterone levels, should increase their intake of egg white protein, while minimizing their intake of fats, in order to optimize their serum testosterone levels. While simple sugars such as glucose and dextrose drinks have been reported to significantly impair testosterone levels, an OJ drink with comparable CHO content appears to have a minimal negative impact on Leydig cell function. Therefore, our pilot data suggest that hypogonadal men should prefer high-quality CHO drinks such as OJ over simple sugars like those contained in a soft drink in order to maintain optimal testicular function. However, we recognize the current study has just examined the acute impact of these dietary interventions on serum testosterone in a small cohort; therefore, larger long-term studies are needed to see if dietary choices can significantly modify testosterone levels over the long term.

It would be interesting to investigate whether these effects are specific to egg white protein and to a less extent to whey protein as well as whether they are driven by specific amino acids or plant-versus-animal protein dietary patterns that are also capable of enhancing serum testosterone. In contrast, any type of fatty meal significantly impaired serum testosterone, and the combination of other nutrients, similar to those found in a McDonald’s meal, was not able to restore testosterone levels. These findings warrant further investigation in larger long-term cohorts, with the aim to increase serum testosterone levels. This may include men wanting to improve their reproductive potential or athletes wishing to improve their athletic performance, not only through testosterone long-term anabolic actions but also through its rapid effects on behavior [41].

While Western dietary patterns have long been associated with reverse associations with testosterone levels, we were surprised to find equal amounts of MUFA and PUFA dietary fats impaired testosterone similarly [17]. This has implications for the Mediterranean dietary patterns. Future intervention studies investigating the mechanism behind the impairment of testosterone production by fats, in contrast to the enhancement of Leydig cell function induced by albumin, are clearly required. Therefore, it may be wise for men wishing to optimize their testosterone levels to avoid a high fat intake, drink liquids such as water or OJ or even consider fasting. As this is the first study to show that egg white protein increased testosterone levels over a 5 h period, further larger chronic studies replicating these findings need to be conducted before dietary recommendations can be made.

## Figures and Tables

**Table 1:** Nutritional composition. CHO: refined carbohydrate, PUFA: polyunsaturated fat, MUFA: monounsaturated fat.

	CHO	Protein (Albumin)	Protein (Whey)	PUFA	MUFA	CHO + Albumin	CHO + PUFA	PUFA + Albumin
Total energy (Kj)	1800	847	739	1930	1872	2647	3730	847
Total fat (g)	<1	<1	<1	52	51	<1	52	<1
Saturated fat (g)	<1	<1	<1	8	8	<1	8	<1
Polyunsaturated fat (g)	<1	<1	<1	31	4	<1	31	<1
Monounsaturated fat (g)	<1	<1	<1	10	36	<1	10	<1
trans Fatty acids (g)	<1	<1	<1	1	0	<1	1	<1
Omega 3 fatty acid (g)	<1	<1	<1	4	0	<1	4	<1
Omega 6 fatty acid (g)	<1	<1	<1	28	4	<1	28	<1
Protein (g)	<1	41	41	1	0	41	1	41
Total carbohydrate (g)	100	<1	<1	6	0	100	100	<1
Sugar (g)	76	<1	<1	0	0	76	76	<1
Fibre (g)	<1	<1	<1	0	0	<1	<1	<1
Sodium (mg)	<5	3	102	5	0	3	5	3

**Table 2:** Demographic, lifestyle and dietary intake data. BMI: body mass index.

	Fasting	CHO	Protein (Albumin)	Protein (Whey)	PUFA	MUFA	CHO + Albumin	CHO + PUFA	PUFA + Albumin	*p* Value
Age (yrs)	37 ± 12.5	33.0 ± 11.0	33.6 ± 11.1	37.0 + 12	32.6 ± 11.1	33.0 ± 10.6	37.0 + 12	37.0 + 12	37.0 + 12	0.913
BMI kg/m^2^	32.3 ± 8.2	31.3 ± 5.2	32.3 ± 4.6	30.7 + 5.1	30.3 ± 5.8	31.3 ± 5.2	30.7 + 5.1	30.7 + 5.1	30.7 + 5.1	0.939
Sleep	6.4 ± 2.3	5.8 ± 3.1	5.7 ± 3.4	6.6 ± 2.4	5.4 ± 3.2	5.4 ± 3.2	6.6 ± 2.4	6.6 ± 2.4	6.6 ± 2.4	0.998
Physical activity	5.5 + 2.1	5.2 ± 6.1	8.0 ± 6.1	5.8 ± 2.1	5.2 ± 1.9	5.2 ± 6.2	5.8 ± 2.1	5.8 ± 2.1	5.8 ± 2.1	0.782
Dietary intake										
Energy (MJ/d)	10.2 ± 3.2	9.7 ± 3.2	9.7 ± 3.2	11.3 + 3.2	9.7 ± 3.2	9.9 ± 3.2	11.3 + 3.2	11.3 + 3.2	11.3 + 3.2	0.921
Total fat (g)	85 ± 15	79 ± 24	84 ± 23 + 24	84 + 13	95 ± 33	79 ± 24	84 + 13	84 + 13	84 + 13	0.134
Saturated fat (g)	29.2 ± 8.0	26.6 ± 11.4	26.6 + 11	29 ± 7	27 ± 11	28 ± 11	29 ± 7	29 ± 7	29 ± 7	0.203
Total protein (g)	92.3 ± 20.2	103 ± 39	103 + 39	93 ± 18	115 + 36	100 + 37	93 ± 18	93 ± 18	93 ± 18	0.222
Total carbohydrates (g)	329 + 108	291 ± 148	290 ± 147	317 ± 123	313 ± 134	294 ± 149	317 ± 123	317 ± 123	317 ± 123	0.965
Sugar (g)	143 ± 101	143 ± 157	141 ± 158	142 ± 87	143 ± 128	140 + 145	142 ± 87	142 ± 87	142 ± 87	0.864
Fibre (g)	23.6 ± 8.1	19 ± 9	19 ± 9	23.4 ± 7.3	26 ± 15	19 ± 10	23.4 ± 7.3	23.4 ± 7.3	23.4 ± 7.3	0.614

**Table 3:** Baseline values for biochemical measures. LH: Luteinizing hormone, FSH: follicle stimulating hormone.

	Fasting	CHO	Protein (Albumin)	Protein (Whey)	PUFA	MUFA	CHO+ Albumin	CHO + PUFA	PUFA + Albumin	*p* Value
Testosterone (nmol/L)	11.2 ± 3.6	13.3 ± 3.1	12.8 ± 2.7	10.8 ± 3.3	15.5 ± 5.4	12.3 ± 2.5	11.0 ± 4.0	10.7 ± 2.5	10.7 ± 2.5	0.018
LH (IU/L)	5.5 ± 1.5	4.7 ± 1.7	5.4 ± 1.9	5.8 ± 2.6	5.0 ± 1.8	5.5 ± 2.4	5.0 ± 2.2	5.2 ± 2.1	4.7 ± 1.3	0.617
Testosterone/LH	2.2 ± 0.8	3.4 ± 2.1	2.6 ± 1.0	2.1 ± 0.8	3.3 ± 1.2	2.5 ± 0.9	2.5 ± 0.8	3.1 ± 1.7	2.2 ± 0.3	0.361
Oestradiol (pmol/L)	123 ± 17	125 ± 48	94 ± 28	48 ± 41	110 ± 30	104 ± 29	117 ± 36	123 ± 30	114 ± 43	0.007
FSH (IU/L)	3.2 ± 1.5	4.2 ± 1.7	3.9 ± 1.6	3.5 ± 1.4	4.5 ± 2.1	3.8 ± 2.0	2.7 ± 0.6	4.4 ± 1.9	3.3+1.5	0.001

## References

[1] (2018). World Health Organisation: Obesity and Overweight. https://www.who.int/news-room/fact-sheets/detail/obesity-and-overweight.

[2] Palmer N.O., Bakos H.W., Fullston T., Lane M. (2012). Impact of obesity on male fertility, sperm function and molecular composition. Spermatogenesis.

[3] Australian Institute of Health and Welfare: A Picture of Overweight and Obesity in Australia 2019. https://www.aihw.gov.au/getmedia/172fba28-785e-4a08-ab37-2da3bbae40b8/aihw-phe-216.pdf.aspx?inline=true.

[4] De Lorenzo A., Noce A., Moriconi E., Rampello T., Marrone G., Di Daniele N., Rovella V. (2018). MOSH Syndrome (Male Obesity Secondary Hypogonadism): Clinical Assessment and Possible Therapeutic Approaches. Nutrients.

[5] Allen N.E., Key J.K. (2007). The effects of diet on circulating sex hormone levels in men. Nutr. Res. Rev..

[6] Hu T.-Y., Chen Y.C., Lin P., Shih C.-K., Bai C.-H., Yuan K.-C., Lee S.-Y., Chang J.-S. (2018). Testosterone-Associated Dietary Pattern Predicts Low Testosterone Levels and Hypogonadism. Nutrients.

[7] Ghanim H., Abuaysheh S., Sia C.L., Korzeniewski K., Chaudhuri A., Fernandez-Real J.M., Dandona P. (2018). Diminished androgen and estrogen receptors and aromatase levels in hypogonadal diabetic men: Reversal with testosterone. Diabetes Care.

[8] Grossmann M. (2018). Hypogonadism and male obesity: Focus on unresolved questions. Clin. Endocrinol..

[9] Hales D. (2002). Testicular macrophage modulation of Leydig cell steroidogenesis. J. Reprod. Immunol..

[10] Tremellen K., Mcphee N., Pearce K. (2017). Metabolic endotoxaemia related inflammation is associated with hypogonadism in overweight men. Basic Clin. Androl..

[11] Tremellen K., McPhee N., Pearce K., Benson S., Schedlowski M., Engler H. (2018). Endotoxin-initiated inflammation reduces testosterone production in men of reproductive age. Am. J. Physiol.-Endoc. Metab..

[12] Gregersen S., Samocha-Bonet D., Heilbronn L.K., Campbell L.V. (2012). Inflammatory and Oxidative Stress Responses to High-Carbohydrate and High-Fat Meals in Healthy Humans. J. Nutr. Metab..

[13] Erridge C., Attina T., Spickett C.M., Webb D.J. (2007). A high-fat meal induces low-grade endotoxemia: Evidence of a novel mechanism of postprandial inflammation. Am. J. Clin. Nutr..

[14] Eslamian G., Amirjannati N., Rashidkhani B., Sadeghi M.-R., Hekmatdoost A. (2012). Intake of food groups and idiopathic asthenozoospermia: A case–control study. Hum. Reprod..

[15] Mendiola J., Torres-Cantero A.M., Grau J., Ten J., Bernabeu R. (2009). Food intake and its relationship with semen quality: A case-control study. Fertil. Steril..

[16] Vujkovic M., de Vries J.H., Dohle G.R., Bonsel G.J., Lindemans J., Macklon N.S., van der Spek P.J., Steegers E.A.P., Steegers-Theunissen R.P.M. (2009). Associations between dietary patterns and semen quality in men undergoing IVF/ICSI treatment. Hum. Reprod..

[17] Gaskins A.J., Afeiche M.C., Hauser R., Williams P.L., Gillman M.W., Tanrikut C., Petrozza J.C., Chavarro J.E. (2014). Paternal physical and sedentary activities in relation to semen quality and reproductive outcomes among couples from a fertility center. Hum. Reprod..

[18] Karayiannis D., Kontogianni M.D., Mendorou C., Douka L., Mastrominas M., Yiannakouris N. (2016). Association between adherence to the Mediterranean diet and semen quality parameters in male partners of couples attempting fertility. Hum. Reprod..

[19] Jurewicz J., Radwan M., Sobala W., Radwan P., Bochenek M., Hanke W. (2018). Dietary Patterns and Their Relationship with Semen Quality. Am. J. Men’s Health.

[20] Attaman J.A., Toth Thomas L., Furtado J., Campos H., Hauser R., Chavarro J.E. (2012). Dietary fat and semen quality among men attending a fertility clinic. Hum. Reprod..

[21] Jensen T.K., Heitmann B.L., Jensen M.B., Halldorsson T.I., Andersson A.-M., Skakkebæk N.E., Joensen U.N., Lauritsen M.P., Christiansen P., Dalgård C. (2012). High dietary intake of saturated fat is associated with reduced semen quality among 701 young Danish men from the general population. Am. J. Clin. Nutr..

[22] Chiu Y.H., Afeiche M.C., Gaskins A.J., Williams P.L., Mendiola J., Jørgensen N., Swan S.H., Chavarro J.E. (2014). Sugar-sweetened beverage intake in relation to semen quality and reproductive hormone levels in young men. Hum. Reprod..

[23] Volek J.S., Gmez A.L., Love D.M., Avery N.G., Sharman M.J., Kraemer W.J. (2001). Effects of a high-fat diet on postabsorptive and postprandial testosterone responses to a fat-rich meal. Metabolism.

[24] Habito R.C., Montalto J., Leslie E., Ball M.J. (2000). Effects of replacing meat with soyabean in the diet on sex hormone concentrations in healthy adult males. Br. J. Nutr..

[25] Meikle A.W., Benson S.J., Liu X.H., Boam W.D., Stringham J.D. (1989). Nonesterified fatty acids modulate steroidogenesis in mouse Leydig cells. Am. J. Physiol. -Endocrinol. Metab..

[26] World Health Organisation (2017). BMI Classifications. http://apps.who.int/bmi/index.jsp?introPage=intro_3.html.

[27] Black A.E. (2000). Critical evaluation of energy intake using the Goldberg cut-off for energy intake:basal metabolic rate. A practical guide to its calculation, use and limitations. Int. J. Obes..

[28] Buysse D.J., Reynolds C.F., Monk T.H., Berman S.R., Kupfer D.J. (1989). The Pittsburgh sleep quality index: A new instrument for psychiatric practice and research. Psychiatry Res..

[29] Baecke J.A., Burema J., Frijters J.E. (1982). A short questionnaire for the measurement of habitual physical activity in epidemiological studies. Am. J. Clin. Nutr..

[30] Tremellen K., Hill A., Pearce K. (2019). Mechanistic insights into the aetiology of post-prandial decline in testosterone in reproductive-aged men. Andrologia.

[31] Martínez-González Miguel A., Gea A., Ruiz-Canela M. (2019). The Mediterranean Diet and Cardiovascular Health. Circ. Res..

[32] Iranmanesh A., Lawson D., Veldhuis J.D. (2012). Glucose ingestion acutely lowers pulsatile LH and basal testosterone secretion in men. Am. J. Physiol.-Endoc. Metab..

[33] Caronia L.M., Dwyer A.A., Hayden D., Amati F., Pitteloud N., Hayes F.J. (2013). Abrupt decrease in serum testosterone levels after an oral glucose load in men: Implications for screening for hypogonadism. Clin. Endocrinol..

[34] Guo Y., Wang A., Liu X., Li E. (2019). Effects of resveratrol on reducing spermatogenic dysfunction caused by high-intensity exercise. Reprod. Biol. Endocrinol..

[35] Cutillas-Tolín A., Mínguez-Alarcón L., Mendiola J., López-Espín J.J., Jørgensen N., Navarrete-Muñoz E.M., Torres-Cantero A.M., Chavarro J.E. (2015). Mediterranean and western dietary patterns are related to markers of testicular function among healthy men. Hum. Reprod..

[36] Fantus R.J., Halpern J.A., Chang C., Keeter M.K., Bennett N.E., Helfand B., Brannigan R.E. (2019). The association between popular diets and serum testosterone among men in the United States. J. Urol..

[37] Rajpathak S.N., Gunter M.J., Wylie-Rosett J., Ho G.Y.F., Kaplan R.C., Muzumdar R., Rohan T.E., Strickler H.D. (2009). The role of insulin-like growth factor-I and its binding proteins in glucose homeostasis and type 2 diabetes. Diabetes-Metab. Res. Rev..

[38] Yoshizawa A., Clemmons D.R. (2000). Testosterone and Insulin-like Growth Factor (IGF) I Interact in Controlling IGF-Binding Protein Production in Androgen-Responsive Foreskin Fibroblasts1. J. Clin. Endocrinol. Metab..

[39] Corona G., Rastrelli G., Monami M., Melani C., Balzi D., Sforza A., Forti G., Mannucci E., Maggi M. (2011). Body Mass Index Regulates Hypogonadism-Associated CV Risk: Results from a Cohort of Subjects with Erectile Dysfunction. J. Sex. Med..

[40] Habito R.C., Ball M.J. (2001). Postprandial changes in sex hormones after meals of different composition. Metabolism.

[41] Wood R.I., Stanton S.J. (2012). Testosterone and sport: Current perspectives. Horm. Behav..

